# Oculomotor and Inhibitory Control in Dyslexia

**DOI:** 10.3389/fnsys.2018.00066

**Published:** 2019-01-08

**Authors:** Thomas D. W. Wilcockson, Diako Mardanbegi, Peter Sawyer, Hans Gellersen, Baiqiang Xia, Trevor J. Crawford

**Affiliations:** ^1^Centre for Ageing Research, Department of Psychology, Lancaster University, Lancaster, United Kingdom; ^2^Sport Exercise and Health Sciences, Loughborough University, Loughborough, United Kingdom; ^3^School of Computing and Communications, Lancaster University, Lancaster, United Kingdom; ^4^School Engineering and Applied Science, Aston University, Birmingham, United Kingdom

**Keywords:** eye tracking, eye movements, dyslexia, inhibition, post-saccadic oscillations, microsaccades

## Abstract

Previous research has suggested that people with dyslexia may have an impairment of inhibitory control. The oculomotor system is vulnerable to interference at various levels of the system, from high level cognitive control to peripheral neural pathways. Therefore, in this work we examined two forms of oculomotor inhibition and two forms of oculomotor interference at high and low levels of the control system. This study employed a prosaccade, antisaccade, and a recent distractor eye movement task (akin to a spatial negative priming) in order to explore high level cognitive control and the inhibition of a competing distractor. To explore low-level control we examined the frequency of microsaccades and post-saccade oscillations. The findings demonstrated that dyslexics have an impairment of volitional inhibitory control, reflected in the antisaccade task. In contrast, inhibitory control at the location of a competing distractor was equivalent in the dyslexic and non-dyslexic groups. There was no difference in the frequency of microsaccades between the two groups. However, the dyslexic group generated larger microsaccades prior to the target onset in the prosaccade and the antisaccade tasks.The groups did not differ in the frequency or in the morphology of the post-saccade oscillations. These findings reveal that the word reading and attentional difficulties of dyslexic readers cannot be attributed to an impairment in the inhibition of a visual distractor or interference from low-level oculomotor instability. We propose that the inhibitory impairment in dyslexia occurs at a higher cognitive level, perhaps in relation to the process of attentional disengagement.

## 1. Introduction

Skilled reading requires a combination of perceptual and phonological skills. Text is segmented into meaningful chunks for the recognition of familiar words which is then translated into a phonological code (LaBerge and Samuels, 1974). This skill is crucially dependent on the fast and efficient ability to focus and shift visual attention rapidly across the relevant text, and to inhibit competing and irrelevant distractors. Developmental dyslexia, which affects 5–17.5% of the population (Shaywitz, 1998; Dmonet et al., 2004), is a reading impairment that is not attributable principally to low intelligence or poor education (e.g., Bradley and Bryant, 1983; Stanovich, 1988; Frith et al., 1995). People with dyslexia have a broad set of symptoms related to cognition, phonological awareness (Vellutino et al., 2004), memory (Liberman et al., 1982), visual processing (Stein, 1990; Pavlidis, 1991; Eden and Zeffiro, 1998; Crawford and Higham, 2001), auditory processing (Tallal, 1980), and attention (Casco et al., 1998; Facoetti et al., 2000). One general theory of dyslexia (Hari and Renvall, 2001) attributes the reading difficulties primarily to a sluggish attentional system. People with dyslexia, according to this view, lack the ability to rapidly distinguish relevant from irrelevant visual information, and are therefore unable to filter distracting signals in the information processing stream. Eden et al. (2004) reported evidence of abnormalities in various aspects of oculomotor control in people with dyslexia, including reduced eye movement stability both during fixations and after saccades (cf. Nyström et al., 2013), and lower vergence amplitudes. The impairment of fixation was found in people with dyslexia irrespective of their phonological ability. An unstable and noisy oculomotor system would contribute to this problem at various processing stages by producing interference as a result of motor instability and visual perturbations. If the eyes are readily distracted and wobble around excessively, this would increase the filtering that is required by the attentional and oculomotor mechanisms. It would not be surprising that high levels of oculomotor interference from instability would contribute to the problem of sluggish attention in dyslexic readers. In this work we focus on four potential sources of oculomotor interference and instability that would impede efficient visual processing and the accuracy of saccadic eye movements during reading: (1) Inhibitory control of an irrelevant saccade (i.e., antisaccade); (2) Accurate target selection in presence of a competing distractor; (3) The over-expression of microsaccades during periods of steady fixation (Bowers and Poletti, 2017); (4) Post-saccadic oscillations that might enhance the retinal slip or motion (i.e., noise) toward the end of a saccade.

### 1.1. Inhibition of Prepotent Saccade (Antisaccade)

The antisaccade task is a commonly used measure of inhibitory control (e.g., Crawford et al., 2005). Neuroimaging studies have indicated that antisaccades are controlled by a network of activation in a fronto-parieto-subcortical network of frontal eye fields (FEFs), supplementary eye fields (SEFs), dorsolateral prefrontal cortex (DLPFC), ventrolateral prefrontal cortex (VLPFC), posterior parietal cortex, supramarginal gyrus (SMG), striatum, thalamus, and cerebellum (O'Driscoll et al., 1995; Sweeney et al., 1996; Müri et al., 1998; McDowell et al., 2002; Matsuda et al., 2004; Tu et al., 2006). Previous research reported that young dyslexics between 7 and 17 years old were impaired on the antisaccade task (Biscaldi et al., 2000). This research supports the hypothesis that dyslexics are impaired in inhibitory control. The antisaccade is a complex executive function that incorporates both sensory and motor distractibility, and consists of multiple cognitive operations including working memory and top down control. Crawford and Higham (2016) demonstrated that inhibitory control and working memory are distinct cognitive operations (Crawford and Higham, 2016). Therefore we predict that antisaccade errors will not be associated with working memory function for the dyslexic participants.

### 1.2. Target Selection and the Inhibition of a Competing Distractor

Converging evidence suggests that people with dyslexia may have an impairment in the inhibition of a visual distractor. Two sources of evidence come from the antisaccade task (Biscaldi et al., 2000) and the Posner cueing task (Facoetti et al., 2003). In the antisaccade task people with dyslexia generated an increase in the frequency of errors in saccades that were directed toward the visual distractor, rather than away from the distractor. Similarly, dyslexics showed faster reaction times compared to controls when a peripheral cue signaled the incorrect location of the target (i.e., invalid cue), but they demonstrated the usual attention benefit for a valid cue in the Posner cueing task (Facoetti et al., 2003).

The antisaccade task and the Posner cueing task have low ecological validity. In everyday life, it is unusual for a cue that appears in one visual field to predict with high reliability the appearance of a target in the opposite field. Neither of these paradigms are analogous to the reading situation where visual attention is required on the target word, while simultaneously inhibiting competing text. In the conventional antisaccade task there is no competing stimulus. Similarly, in the Posner cueing task at the time of the attentional cue there is no competition between a target and a distractor. These tasks may therefore require the ability to disengage from a prepotent target that has captured attention, rather than the ability to inhibit or suppress a competing distractor. It is this latter process that appears to be more directly relevant to the reading task, where target words are selected in each fixation from competing, alternative words. Therefore in this study we have turned to the inhibition of recent distractor (IRD) previously used by Crawford et al. (2005) and Donovan et al. (2012). We contrast performance in the recent distractor task and the conventional antisaccade task in dyslexic and normal readers.

The IRD task does not depend on an eye movement away from a visual target nor a “misleading” visual cue, which misleads the participant about the impending location of the target. Here, participants are presented with a sequence of two critical displays. In one display a red target is presented together with a green distractor. This is followed by a display with a new red target presented in isolation at one of three locations (with respect to the previous display). The singleton target is presented either at the location of the recent target (target-target: TT), the location of the recent distractor (target-distractor: TD), or a new location (target-neutral: TN). Participants are instructed to fixate the target in both displays and to ignore the green distractor. Crawford et al. (2005) demonstrated that saccadic latencies to the singleton target were reliably slowed for a target that appeared at the location of a recent distractor, showing that attention has a dual function: facilitation of eye movements to the target and inhibition of eye movements to a distractor (Crawford et al., 2005). In this work we investigate whether or not this inhibition of a distractor is present or weakened in dyslexic readers.

Inhibitory control is clearly not a unitary concept, and has many different forms that can be dissociated at various levels of the visuomotor control networks. Therefore it cannot be assumed that the antisaccade task and the recent distractor task target the same inhibitory mechanisms, indeed the dyslexia findings here demonstrate that they do not. In the antisaccade task the eye movement is directed away from the target. This motor requirement is absent from the recent distractor task. In contrast to the antisaccade task, the presence of a distractor that competes with the target is essential for the generation of “distractor inhibition,” and distinguishes this clearly from the antisaccade task. This is a critical factor for inhibition at the location of a distractor has been shown in many negative priming studies. Importantly, our previous research has clearly demonstrated that the antisaccade task is not sufficient to generate the “spatial inhibition at the location of distractor” that we find in the recent distractor task (Crawford et al., 2005). Donovan et al. (2012) demonstrated that the presence of distractor in the probe display as well as the prime display is also require for object inhibition (Donovan et al., 2012). Spatial inhibition at the location of a distractor is enhanced in the presence of a competing target. Donovan et al. (2012) showed that in a condition where there is no competing distractor in the probe display, no negative priming for visual objects were generated (or inhibition at the location of the distractor). When a competing target was introduced together with a distractor, inhibition was generated at that location. So this form of inhibition is specific to these tasks, and is not generated in the antisaccade task. The antisaccade requires a motor signal to move the eyes in the opposite direction, not a signal to suppress the target itself.

### 1.3. Are Microsaccades Over-expressed in Dyslexic Readers?

In recent years an interest in the phenomena of microsaccades has resurfaced, partly driven by the availability of modern user-friendly eye-tracking technology. However, to our knowledge microsaccades have not yet been explored as a potential causal factor in dyslexia. Microsaccades are miniature versions of larger saccades that reposition the visual image within the foveal region (i.e., with 1° of visual angle or substantially less). They are common in healthy populations but occur with greater frequency or larger amplitudes (or with other characteristics) in neurological disorders (Abadi and Gowen, 2004; Kapoula et al., 2014). Their precise function is controversial, although altered fixational eye movements are found in disorders of cognition, including attention and working memory (Martinez-Conde et al., 2013). Microsaccades have been regarded as a noise feature of the oculomotor system or as “involuntary” movements that are necessary for preventing neural adaptation and the perceptual fading experienced in the complete absence of retinal image motion (Martinez-Conde et al., 2006). However, there is growing evidence that microsaccades serve a similar function to larger saccades as they play an important role in enhancing visual acuity and the allocation of visual attention in perceptual tasks (Poletti et al., 2013). Microsaccades and standard saccades are apparently controlled by the same neural structures (Havermann et al., 2014) and follow common motor characteristics (Hafed and Krauzlis, 2012). The over expression of microsaccades would clearly be counter-productive during a reading task. Indeed, the frequency of microsaccades is reduced during normal reading in comparison to non-reading visual fixations (Bowers and Poletti, 2017). Remarkably, in normal readers microsaccades follow a systematic pattern. They occur close to the end of words, and are predominantly regressive, within-word fixations. The nature of microsaccades in dyslexic readers is unknown. For example, it is unclear whether or not there is an excess of microsaccades that could potentially contribute to the perturbation of visual processing in dyslexic readers. Therefore in this work we contrasted microsaccades between normal and dyslexic readers.

### 1.4. Visual Interference From Post-saccadic Oscillations in Dyslexia?

The movement of the eyes do not come to an abrupt stop at the end of a saccade, or immediately on the arrival at the target word. There is a characteristic eye wobble at the end of a saccade, that is known as a post-saccadic oscillation (PSO) that appears to originate from a combination of sources (Eizenman et al., 1984; Nyström et al., 2013) including the mechanics of the eye, the cornea, and the iris muscles. Therefore the amplitude and specific feature of PSO is partly influenced by video-based eye-tracking methodologies. Note that PSO or what was referred to as “dynamic overshoot” was reported by Bahill et al. (1975) using an infra-red limbus eye-tracker. Thus PSO cannot be an artifact of the video-based eye-tracking systems (e.g., EyeLink). Changes in the PSO signal are sensitive to the relative displacement of the lens and the cornea in the Dual Purkinge devices (Kimmel et al., 2012), whilst during and after movement the structural changes in the iris during saccade are detected in video-based eye trackers that are centered on the pupil (Nyström et al., 2013). So the nature of PSO signal needs to be considered in light of the specific eye-tracking methodology. However, it is clear that in addition to PSO and microsaccades there are other potential visual perturbations that can arise as a consequence of oscillations at the end of the saccade movement itself. These visual perturbations would be caused by the retinal slip of the image on the retina and mild oscillopsia. Further, post saccade oscillations may also delay the processing of visual information. It is currently unknown whether these sources of visual perturbations contribute to the reading disorders of dyslexic readers. This is particularly important given the frequent reports of visual motion phenomena in dyslexic readers.

## 2. Methods

### 2.1. Participants

Thirty three participants were recruited: 18 dyslexic (8 male, 10 female; mean age = 19.81 years, range = 18–22, *SD* = 1.05) and 15 non-dyslexic controls (5 male, 10 female; mean age = 20.47 years, range = 18–27, *SD* = 2.59). All participants were university students. All participants had normal or corrected visual acuity (assessed with the Snellen chart), and intact color vision according to the Ishihara test (Clark, 1924). Dyslexic participants were recruited with the help of the Lancaster University Disability Office. The dyslexic participants had all been previously diagnosed with dyslexia by an educational psychologist and volunteered to take part in the study. Controls were obtained by offering psychology students subject pool credit. Table 1 shows the cognitive assessment scores (described below) of the two groups. As can be seen in the table, the dyslexics did not differ significantly from the controls in terms of working memory, Ravens matrices, and WRAT maths. Full ethical approval was granted by the Department of Psychology Research Ethics Committee.

**Table 1:** Cognitive assessment scores (means and SD) of the dyslexics and controls participants.

**Assessments**	**Dyslexic**	**Controls**	***p***
Working memory score	27.0 (6.6)	21.6 (9.5)	>0.05
CTOPP phonological memory	115.8 (7.8)	100.3 (9.0)	< 0.05
CTOPP rapid naming	94.4 (14.1)	79.5 (16.1)	< 0.05
CTOPP elision SS	10.7 (1.2)	9.2 (2.0)	< 0.05
Ravens/36	24.1 (5.7)	22.0 (6.4)	>0.05
WRAT reading	108.1 (6.6)	102.3 (8.1)	< 0.05
WRAT spelling	113.0 (11.2)	98.3 (10.3)	< 0.05
WRAT math	104.1 (13.2)	99.9 (17.6)	>0.05

*Significant group effects (p < 0.05) are shown by the p column. CTOPP, Comprehensive Test of Phonological Processing (Bruno and Walker, 1999); WRAT, Wide Range Achievement Test 4 (Wilkinson and Robertson, 2006); Ravens matrices non-verbal IQ measure (Raven, 2003)*.

### 2.2. Procedure

The eye movement experiments were conducted in the eye movement laboratory at Lancaster University. An EyeLink Desktop 1000 (SR Research Ltd., Ontario, Canada) at 500 Hz and Experiment Builder Software were used to control the stimulus events. Participants sat 55 cm away from the screen and used a chin rest. Each participant completed three eye-tracking tasks; prosaccade, antisaccade, and a recent distractor task (see below). Participants were also assessed on a battery of standard assessments of cognitive impairment in dyslexia: the phonological memory, rapid naming, and Elision Standard Score sections of the Comprehensive Test of Phonological Processing (CTOPP: Bruno and Walker, 1999), the reading, spelling, and math sections of the Wide Range Achievement Test 4 (WRAT4: Wilkinson and Robertson, 2006); Ravens matrices for estimation of non-verbal IQ (Raven, 2003). Working memory capacity was assessed using the Daneman and Carpenter (1980) sentences (Daneman and Carpenter, 1980) drawn from Friedman and Miyake (2004). Participants read aloud a sentence that appeared on the screen. The sentence was then replaced by a single key word (presented in a distinctive purple font), which again the participants read out loud. After each block of sentences the participant was instructed to recall as many of the key words as possible by entering the key words in a series of boxes on the computer screen. The first block of trials comprised 5 sets of 2 sentences, a second block comprised of 5 sets of 3 sentences, and the final block comprised of 5 sets of 4 sentences. Working memory span was determined by the total number of words that were correctly recalled in the appropriate order.

### 2.3. Prosaccade Task (PS)

Each participant completed 60 gap trials in the prosaccade task. Each trial was preceded by a 1 s instruction screen. A central fixation was displayed in white on a black background. The white stimulus had a luminosity of 8–9 cd/m^2^ whilst the black background had a luminosity of 0.4–0.8 cd/m^2^. This was displayed for 1 s and participants were instructed to look at this. A blank screen was then displayed for 200 ms. The saccade target (in green) was then presented in a random order 4° away from where the fixation target had been either on the left or right side for 2 s. Participants were instructed to make horizontal eye movements toward the target as quickly and as accurately as possible. The white fixation target and green saccade target were circular and each measured 15 × 15 pixels; 0.83 visual degrees in diameter.

### 2.4. Antisaccade Task (AS)

The parameters in the antisaccade task were the same as the prosaccade task. However, participants were instructed to fixate at the central point then generate the saccade to the opposite position of the screen as soon as the target appeared.

### 2.5. Inhibition of Recent Distractor Task (IRD)

**Figure 1:**
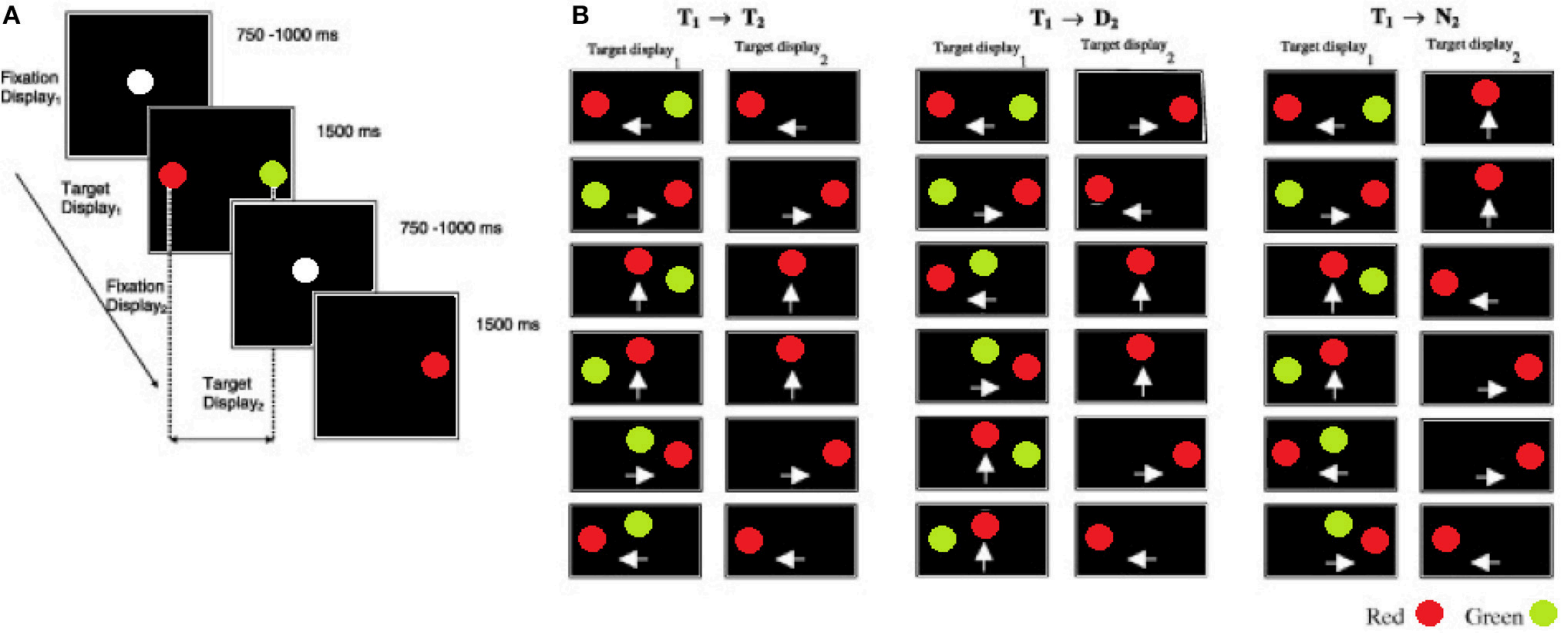
The sequence and the timings of the eye movement displays in the inhibition of recent distractor task (IRD). **(A)** Fixation display1 shows the fixation target at the start of a trial. Participants were instructed to fixate on the red target and to ignore the green distractor in target display1. This was followed by fixation display2. Participants fixated on the lone target in target display2. **(B)** The target–distractor conditions of the experiment. On the *T*1 → *T*2 trials, the target (red) was presented at the same location in target display1 (T1) and target display2 (T2). On the *T*1 → *D*2 trials, the target in target display2 was presented at the location of the distractor in the target display1. On the *T*1 → *N*2 trials the target in the target display2 was presented at a new location, that was not previously occupied by the target or distractor. The white arrows indicate the direction of saccadic eye movement either left, right, or up from the centre-point of the screen).

Each participant began the inhibition of recent distractor task with a practice session of 24 trials followed by 120 mixed, random trials. An IRD trial began with the onset of a white fixation point at the center of a black display (see Figure 1; fixation display1) for a period of 750–1000 ms; this time was randomized to prevent anticipatory responses. The fixation point was then removed and immediately followed by a red target and a green distractor (target display1) presented simultaneously for 1500 ms at 4° away from the fixation point. In contrast to the pro and anti saccade task, the IRD task does not include a temporal blank gap, between the fixation and target displays. Participants were instructed to look at the red target as quickly and as accurately as possible and to ignore the green distractor. Once the target display1 was removed the fixation point re-appeared for a randomized interval of 750–1000 ms (fixation display2). Finally, participants were instructed to fixate on a single red target (target display2) that was presented for 1500 ms. The stimulus onset asynchrony (SOA) between the target display1 and target display2 was 2250–2500 ms. A blank interval of 3500 ms elapsed before the next trial commenced. The spatial configuration and mapping of the target display1 (recent) and target display2 (new) was a key manipulation (see Figures 1A,B). The target display1 configurations were randomly selected from one of the 18 displays illustrated in Figure 1. The pairings of target display1 and target display2 generated three types of trials: (1) on the *Target*→*Target* (*T*1 → *T*2) trials the display2 target was presented at the location that was previously occupied by the recent target in display1. (2) On the *Target*→*Distractor* (*T*1 → *D*2) trials the display2 target was presented at the location previously occupied by the recent distractor in display1. (3) On the *Target*→*New* (*T*1 → *N*2) trials the display2 target appeared at a new location, not previously occupied by either the target or the distractor in display1. On 50% of the trials the target location was repeated in display2 (i.e., *T*1 → *T*2 trials) and on 50% of trials the target location was different to the display2 target (25% *T*1 → *N*2 +25% *T*1 → *D*2), to ensure that the target location in display1 was non-informative. Therefore, within a complete block of trials each *T*1 → *T*2 was repeated 10 times, while a given *T*1 → *D*2 and *T*1 → *N*2 was repeated five times. These probabilities were chosen in order to encourage a prepotent *T*1 → *T*2 response. TT, TD, and TN mean saccade reaction times were computed for each participant.

## 3. Results

Table 1 shows the cognitive assessment scores of the two groups. Unsurprisingly for a dyslexia group, this sample revealed a substantial impairment in phonological skills, including CTOPP phonological memory, the Elesion measure of the phonological ability and rapid naming, and in the WRAT reading and writing scores. Ravens matrices IQ and WRAT Math scores did not differ significantly between the two groups.

### 3.1. Prosaccade (PS) and Antisaccade Task (AS)

**Figure 2:**
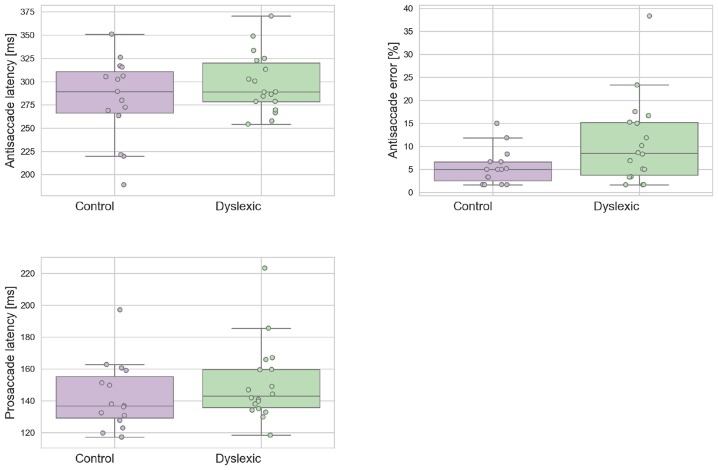
Dyslexic and control mean prosaccade latencies, antisaccade errors, and antisaccade latencies. Error bars show the standard errors.

A Shapiro-Wilk test was used to test for normality on the latency variables. The analyses revealed that neither the dyslexic PS (*p* = 0.543) and AS (*p* = 0.710) or control PS (*p* = 0.127) and AS (*p* = 0.274) violated the assumption of normality distribution. Figure 2 shows the mean prosaccade and antisaccade latencies and standard deviations for the dyslexics and controls groups for prosaccade latencies. The dyslexia group generated a significantly higher proportion of antisaccade errors (*mean* = 13.81;*SD* = 10.57) in comparison to the control group (*mean* = 7.56;*SD* = 5.56) in antisaccade errors [*t*_(31)_ = 2.063; *p* = 0.048; effect size = 0.74]. There was no effect of group for mean prosaccades latencies [*t*_(31)_ = 0.961; *p* = 0.344; effect size = 0.34] or mean antisaccades latencies [*t*_(30)_ = 0.461; *p* = 0.154; effect size = 0.50]. The WM Scores did not correlate significantly with prosaccade latencies in dyslexics [*r*_(15)_ = 0.104; *p* = 0.692] or controls [*r*_(13)_ = –0.236; *p* = 0.397]. Neither was there a significant correlation between WM scores and antisaccade latencies in dyslexics [*r*_(15)_ = –0.261; *p* = 0.312] nor controls [*r*_(13)_ = 0.141; *p* = 0.616] or WM score and antisaccade errors in dyslexics [*r*_(15)_ = 0.103; *p* = 0695] nor controls [*r*_(13)_ = 0.045; *p* = 0.873]. This is consistent with the independence hypothesis of working memory and inhibitory control (Crawford and Higham, 2016). Prosaccade latencies did not correlate significantly with antisaccade latencies in dyslexics [*r*_(15)_ = 0.051; *p* = 0.846] and controls [*r*_(13)_ = 0.374; *p* = 0.170]. Prosaccade latencies did not correlate significantly with antisaccade errors in dyslexics [*r*_(15)_ = –0.247; *p* = 0.340] or controls [*r*_(13)_ = 0.220; *p* = 0.430].

### 3.2. Inhibition of a Recent Distractor (IRD) Task

**Figure 3:**
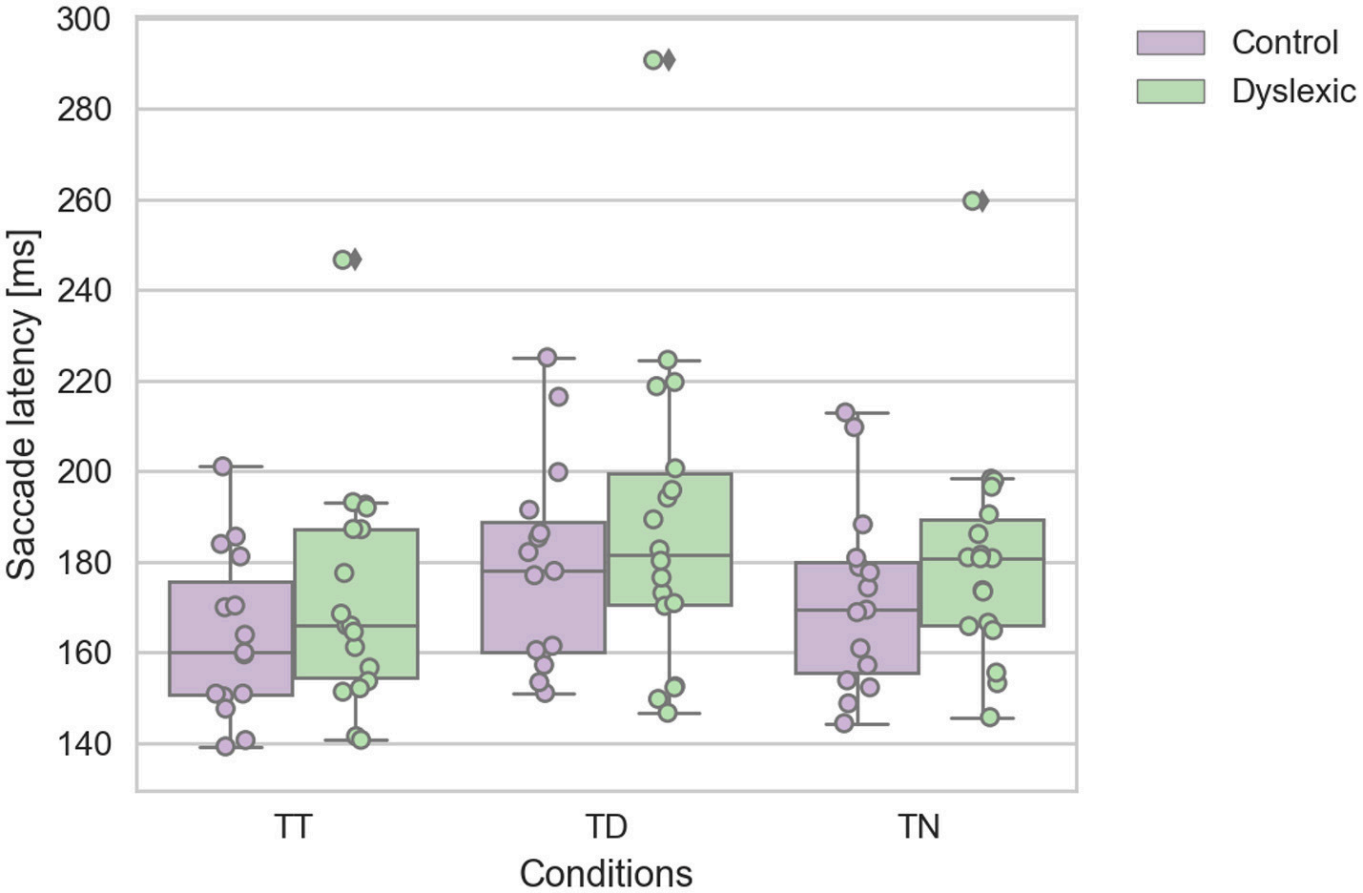
Dyslexic and control mean saccade reaction time for target-target (TT), target-neutral (TN), and target-distractor (TD) trials. Error bars show the standard errors.

A Shapiro-Wilk test was again used to test for the normality distribution on the IRD latency variables. This analysis revealed that the control TT (*p* = 0.475), TD (*p* = 0.223), and TN (*p* = 0.286) latencies were not in violation of normality. Nor were the dyslexic TT (*p* = 0.084), and TD (*p* = 0.207) latencies. However, the TN (*p* = 0.028) latencies were found to be in violation of normality assumption. We identified one dyslexic participant who emerged as an statistical outlier which caused this deviation from normality in the TN condition. We reanalyzed the data with this outlier removed, which then satisfied the assumptions of normality. This reanalysis replicated the findings below, therefore we report the findings with the complete dataset including the one outlier. A repeated-measures ANOVA was conducted on the saccadic mean latencies as the within-subjects factor of the target-distractor configuration (TT; TD; TN) and group factor (dyslexic vs. non-dyslexic control) as the between-subjects factor. This analysis revealed a significant main effect of target-distractor configuration [*F*_(2, 62)_ = 29.032; *p* < 0.0005; effect size = 0.484]. The saccadic mean latencies were slowed on TD trials in comparison to TT & TD trials (see Figure 3). There was no significant effect of group [*F*_(1, 31)_ = 1.038; *p* = 0.316; effect size = 0.032]. There was no interaction effect of group and target configuration [*F*_(2, 62)_ = 0.083; *p* = 0.920; effect size = 0.003]. The effect of target-configuration was evident and of a similar magnitude across both groups (see Figure 3).

## 4. Microsaccades

**Figure 4:**
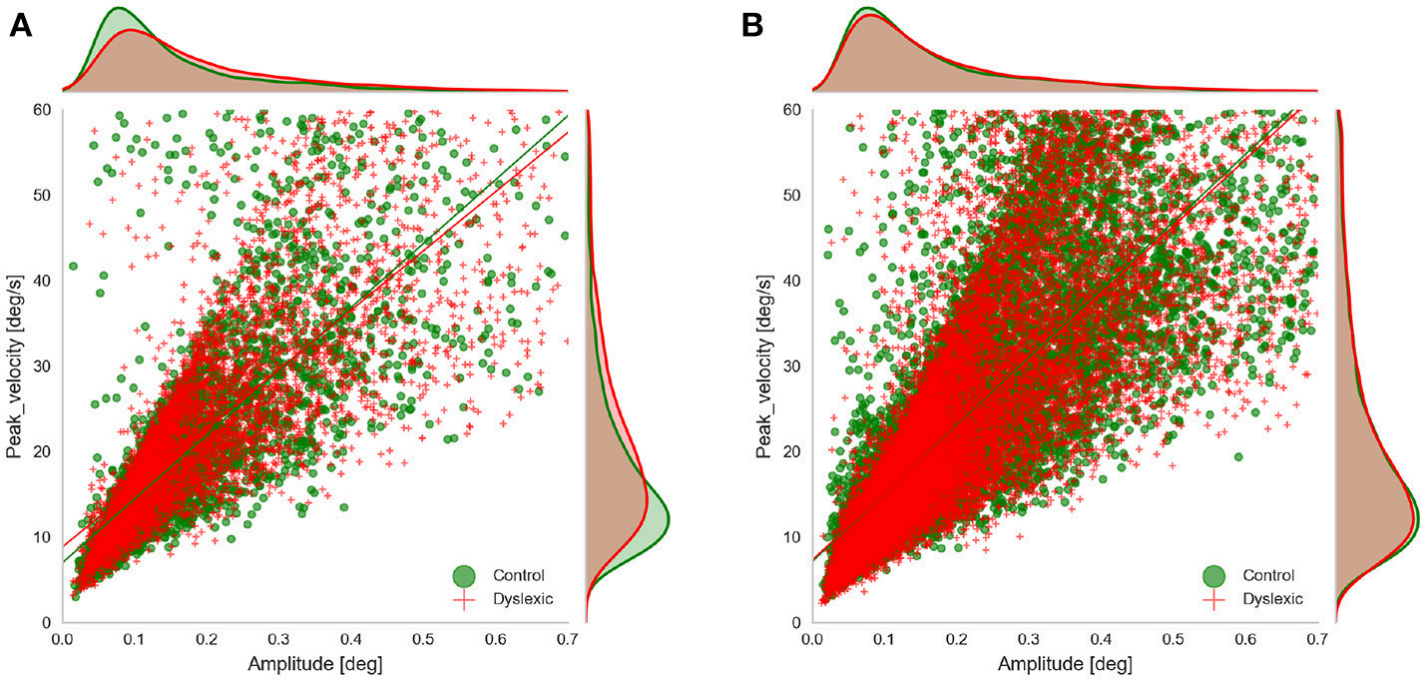
Main sequence of the microsaccades showing microsaccades of both groups obtained in **(A)** AS experiment, and **(B)** RD experiment, during fixations prior to target onset.

We examined the rate of microsaccades per trial across the two groups. We extracted the microsaccades made during the fixations before target onset in all three experiments. Microsaccades were extracted using the approach proposed by Engbert and Kliegl (2003). Their algorithm identifies the microsaccades as “outliers” in two-dimensional velocity space. They define outliers as segments in 2D velocity space that lie outside a threshold (an ellipse in 2D velocity space) which is defined based on a multiple of the standard deviation of the velocity distribution. We applied the microsaccade detection algorithm only to segments of raw gaze points identified as fixations. In fact we did not compute the velocity ourselves and only used the velocity signals as detected by Eyelink. We detected candidate microsaccades based on monocular eye tracking data and filtered those candidates that had the amplitude and duration outside the range of (0.01, 0.7) and (5 ms, 40 ms) respectively, based on the findings of Engbert and Kliegl (2003) and Martinez-Conde et al. (2006). Since our microsaccades were extracted using only monocular data we wanted to check the validity of our microsaccade detection by looking at the main sequence of the detected microsaccedes (Figure 4). This also allowed us to further compare the main characteristics of microsaccades (the amplitude and peak velocity) across the two groups in AS and RD experiments. We do not show the main sequence of the PS experiment because it was very similar to the data from the AS experiment. To facilitate this comparison and to see if any of these two characteristics deviates across the two groups, we fitted a linear regression model to the main sequence of each of the groups and compared the results. The results of the linear regression showed that the main sequence of the microsaccades was following the line *vel*_*peak*_ = 74.7*amp*+7.0(*r* = 0.79, *p* = 0.0) for the Control group and *vel*_*peak*_ = 69.3*amp*+8.8(*r* = 0.78, *p* = 0.0) for the Dyslexic group in the AS task. The result of the linear regression was *vel*_*peak*_ = 72.5*amp*+7.5(*r* = 0.78, *p* = 0.0) for the Control group and *vel*_*peak*_ = 69.53*amp*+8.3(*r* = 0.80, *p* = 0.0) for the Control in the PS experiments. The result of the linear regression was *vel*_*peak*_ = 79.5*amp*+7.1(*r* = 0.81, *p* = 0.0) for the Control group and *vel*_*peak*_ = 78.15*amp*+7.3(*r* = 0.80, *p* = 0.0) for the Control in the RD experiments. As we can also see in the figure, the slope and the intercept of the fitted lines were quite similar in both groups, and overall, they resemble the findings of Engbert and Kliegl (2003).

We further compared the amplitude and peak velocities by taking the mean of all the observations belonging to the same person and comparing the means across the two groups. We did a t-test on the mean amplitude and the mean peak velocity in all three experiments. We found no significant difference between the amplitude [*t*_(31)_ = –0.63; *p* = 0.54] and peak velocities [*t*_(31)_ = –0.38; *p* = 0.71] of the two groups in the IRD experiment. However, in the AS experiment, the mean amplitude [*t*_(31)_ = –4.06; *p* = 0.00; $Mean_{Control}=0.15^{{}^{\circ}},Mean_{Dyslexic}=0.19^{{}^{\circ}}$] and mean peak velocity [*t*_(31)_ = –3.06; $\text{p}=0.00;Mean_{Control}=18.54^{{}^{\circ}}/\text{s},Mean_{Dyslexic}=22.42^{{}^{\circ}}/\text{s}$] of the control group was significantly lower than the dyslexia group. We observed no significant difference between the peak velocities of the two groups in the PS experiment. The mean amplitude was still lower for the control group in the PS experiment [*t*_(31)_ = –2.22; *p* = 0.03].

We compared the average number of microsaccades per trial across our control and dyslexic groups. On average about 3.5 microsaccades per trial were observed in both groups in all experiments. We found no significant difference between the two groups (*p* > 0.05) in terms of the average number of microsaccades per trial in any of the experiments.

## 5. Post-Saccadic Oscillations

**Figure 5:**
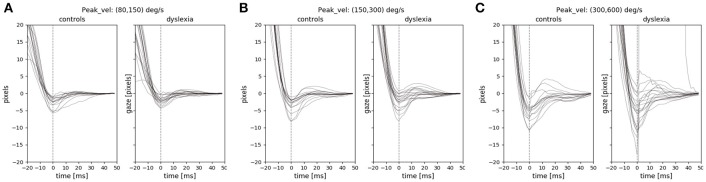
The PSO signals of the two groups for three different saccade peak velocities **(A)** 80–150 **(B)** 150–300 **(C)** 300–600. The PSO signals within each range of peak-velocity are grouped for each subject, and therefore, each signal in the figure represents the median of multiple signals.

We examined the instability and oscillations at the end of each saccade (the PSOs) between the two groups. We used the PSOVIS software (Mardanbegi et al., 2017) to extract and align the PSO signals from the eye movement data of the RD experiment based on the saccade detection performed in the Eyelink tracking software. The PSOVIS software extracts the oscillations along the direction of each saccade. Thus, each PSO signal is a time series signal representing the saccade changes measured in pixels (along the main direction) over time. The minimum peak of each oscillation is then defined as the first critical point of the signal that happens after the maximum velocity. All the signals are then temporally aligned based on their minimum peak which is actually the first overshoot of the PSOs. Each signal is then shifted along the spatial axis such that all the signals converge at zero (see Figure 5).

We focused on the first screen of RD experiment because it was a task that involved inhibitory control and the saccades were made toward clear end-point targets in different directions. Previous studies have shown the effect of saccade peak-velocity on the PSO (Nyström et al., 2016), therefore we looked at the PSO signals separately for different ranges of peak velocities. Figure 5 shows the median PSO signals for three different ranges of peak velocities from 80 to 600°/s. Each signal in the figure represents the median of all PSOs of an individual subject that belong to saccades with peak velocities within a certain range. As we see in the figure, the size of the PSO signals were not very different between the two groups. We compared the amplitude of the PSO signals between the two groups when the amplitude of each individual PSO signal is defined as the distance between the first occurrence of the minimum and the first occurrence of the maximum value of the signal within the interval of 0–40 ms (similar definition was used by Mardanbegi et al., 2018). A *t*-test was conducted to compare the amplitudes of median PSO signal (with peak velocities between 80 and 600°/s) and the result [*t*_(31)_ = –0.26, *p* < 0.8] indicated no significant difference between the mean of PSO amplitude of the control group (*M* = 1.29°, *SD* = 1.23°) and the dyslexia group (*M* = 1.42°, *SD* = 1.51°).

## 6. Discussion

One influential theory of dyslexia claims that the disorder is caused by a sluggish attention system, that involves deficiencies in the inhibition of irrelevant sensorimotor control. Inhibitory control is not a unitary concept, therefore in this work we examined two forms of oculomotor inhibition and two forms of oculomotor interference at high and low levels of the control system. We replicated the reported impairment of antisaccade control in people with dyslexia. Phonological working memory span was reduced in the dyslexic readers, but was not correlated with the frequency of antisaccade errors. This is consistent with the idea that working memory may be associated with inhibitory control but can be dissociated from it Crawford et al. (2011), Crawford and Higham (2016). How then might the dyslexia impairment in the AST be explained if it is not caused by either direct deficits of working memory or distractor suppression? The sluggish attentional theory argues that people with dyslexia are slowed in the shifts of visual attention, which would impede the efficient and rapid processing in the flow of information. For example, Hari et al. (1999) demonstrated, using an attentional blink task, that dwell time was increased by 30% in dyslexic, compared to normal readers. In AST the highly salient singleton target, could lead to a slower attentional disengagement in the dyslexic readers. According to RACE models of the AST (Crawford et al., 2011) a slowed disengagement will cause an increase in the frequency of errors.

Visual sensitivity is not determined simply by the proximity of the stimulus image to the fovea on the retina. The spatial modulation of visual attention determines the gain of activity of neurons in the visual cortex (e.g., Smith et al., 2000). This has been demonstrated across various visual operations including perception of velocity, luminance, and color discrimination. Importantly, increased activation of visual cortex is accompanied by general suppression of neuronal activity representing the surrounding visual field (see Smith et al., 2000). Thus the efficient modulation of selective attention is characterized by the dual properties of increased gain for the visual target and surrounding inhibition of the competing distractors. The current study has demonstrated that the inhibition of visual distractors are apparently preserved in dyslexic readers. Spatial inhibition at the location of distractor was measured using the inhibition of the recent distractor paradigm (Crawford et al., 2005). Interestingly, dyslexia readers demonstrated the normal pattern of distractor inhibition. Apparently, the inhibition deficits of people with dyslexia cannot be attributed to spatially-derived location encoding.

Despite the recent growth in work on microsaccades there has been little work in the context of reading behavior or dyslexia. One important study revealed a highly organized pattern of microsaccades in normal readers. For English readers microsaccades are predominately elicited at the end of words (or sentences), and they tend to be regressive, taking the eye back toward the previous word (see Bowers and Poletti, 2017). They appear to serve a similar function to large saccades and reflect the shifts of vision attention within the target word. Microsaccade frequency appears to be preserved in people with dyslexia. A problem of excessive or intrusive microsaccades clearly cannot explain the reading and visumotor disturbances in dyslexia. However, it is worth noting that microsaccades were of larger amplitude and peak velocity in the AS condition, and were generated closer to the target onset. The impact of these subtle effects are unclear but warrant further work.

Finally, we investigated the post-saccadic oscillations to determine whether this oculomotor phenomenon might account for the reported visual perturbations and attention difficulties of dyslexic readers. Our findings currently rule this out as an explanatory factor.

Dyslexia remains a mysterious and complex disorder with both cognitive and motor features. The dyslexic group revealed a clear impairment on phonological memory and inhibitory antisaccade errors as previously shown (Biscaldi et al., 2000). The antisaccade impairment cannot be attributed to working memory as this was well preserved in this sample, although phonological memory was reduced (cf. Crawford et al., 2011; Crawford and Higham, 2016). A top-down “sluggish” attentional signal might account for the increased antisaccade errors. Conceivably the neural signal to inhibit the prepotent saccade may be slow in arriving at the inhibitory centers in the FEF, DLPFC and fixation cells of the superior colliculus in dyslexic readers. However, the fundamental characteristics of the prosaccadic eye movements were preserved. These findings demonstrate that people with dyslexia do not suffer from a difficulty in selecting a salient target in the presence of a competing distractor. The neural signature of inhibition of the distractor was detected in the slowed response toward target presented at that location on the subsequent display screen. This inhibition was equivalent to that seen in the normal readers. The visual disturbances and the reading difficulties that are experienced by dyslexic readers clearly are not a consequence of oculomotor noise generated by excess microsaccades or post-saccadic oscillations. This work confirms that inhibitory control is not a unitary concept and that it is important to use a range of inhibitory control tasks to isolate the different types and levels of inhibition and potential interference in the oculomotor system.

## Ethics Statement

This study was carried out in accordance with the recommendations of Lancaster University, Faculty of Science and Technology Ethics Committee. The protocol was approved by the Faculty of Science and Technology Ethics Committee. All subjects gave written informed consent in accordance with the Declaration of Helsinki.

## Author Contributions

The original concept for the study was initiated by TC. TW and DM conducted the statistical analyses. TW produced the first draft of the paper. All authors approved the final submission.

### Conflict of Interest Statement

The authors declare that the research was conducted in the absence of any commercial or financial relationships that could be construed as a potential conflict of interest.
